# Projection Stereolithographic Fabrication of BMP-2 Gene-activated Matrix for Bone Tissue Engineering

**DOI:** 10.1038/s41598-017-11051-0

**Published:** 2017-09-12

**Authors:** Hang Lin, Ying Tang, Thomas P. Lozito, Nicholas Oyster, Robert B. Kang, Madalyn R. Fritch, Bing Wang, Rocky S. Tuan

**Affiliations:** 1 Center for Cellular and Molecular Engineering, University of Pittsburgh, Pittsburgh, Pennsylvania 15219 USA; 2 Molecular Therapy Laboratory, University of Pittsburgh, Pittsburgh, Pennsylvania 15219 USA; 3 Stem Cell Research Center, Department of Orthopaedic Surgery, University of Pittsburgh, Pittsburgh, Pennsylvania 15219 USA; 4 Department of Bioengineering, University of Pittsburgh, Pittsburgh, Pennsylvania 15219 USA

## Abstract

Currently, sustained *in vivo* delivery of active bone morphogenetic protein-2 (BMP-2) protein to responsive target cells, such as bone marrow-derived mesenchymal stem cells (BMSCs), remains challenging. *Ex vivo* gene transfer method, while efficient, requires additional operation for cell culture and therefore, is not compatible with point-of-care treatment. In this study, two lentiviral gene constructs – (1) Lv-BMP/GFP, containing human *BMP-2* and *green fluorescent protein (GFP)* gene (BMP group); or (2) Lv-GFP, containing *GFP* gene (GFP group) – were incorporated with human BMSCs into a solution of photocrosslinkable gelatin, which was then subjected to visible light-based projection stereolithographic printing to form a scaffold with desired architectures. Upon *in vitro* culture, compared to the GFP group, cells from BMP group showed >1,000-fold higher BMP-2 release, and the majority of them stained intensely for alkaline phosphatase activity. Real-time RT-PCR also showed dramatically increased expression of osteogenesis marker genes only in the BMP group. 3.5 months post-implantation into SCID mice, the micro-computed tomography imaging showed detectable mineralized areas only in the BMP group, which was restricted within the scaffolds. Alizarin red staining and immunohistochemistry of GFP and osteocalcin further indicated that the grafted hBMSCs, not host cells, contributed primarily to the newly formed bone.

## Introduction

Each year, more than 1 million bone fracture patients are hospitalized in the United States, and 5–10% of them present delayed healing or nonunion, representing a major clinical challenge in orthopaedic surgery^1^. Nonunion, left untreated, causes pain, limits mobility, and increases healthcare cost^2^. So far, no effective medications are available to directly stimulate intrinsic bone regeneration; a surgical intervention is often required. For example, autologous bone graft, a procedure that involves harvesting normal bone tissues from healthy area and implanting into defect sites, has been frequently used and is considered as the “gold standard” for nonunion fractures. However, this treatment has several drawbacks, such as donor site morbidity, limited tissue availability, and molding challenges.

Tissue engineering, an emerging biomedical technology that aims to produce replacement tissues *in vitro*, offers exciting promise to overcome the obstacles of using native tissues. In principle, cell-based bone tissue engineering involves three basic components: osteogenic cells, osteoconductive scaffolds, and osteoinductive growth factors^3^. These components can be used individually or in combination. Irrespective of the approach, it is generally assumed that a strong osteoinductive cue is critical for successful bone repair. Growth factors known to be important for bone development and healing, such as recombinant bone morphogenetic protein-2, 7 (BMP-2, 7), platelet-derived growth factor (PDGF), and vascular endothelial growth factor (VEGF), have been applied and investigated in the context of bone regeneration, among which BMP-2 is used most widely because of its high efficacy in stimulating osteogenesis^4^. A systematic review reported that addition of BMP-2 significantly increased the efficacy of conventional intervention for the healing of acute open tibia fractures^5^, and a 44% reduction in bone nonunion was also observed^6^.

Technically, there are 2 main avenues for BMP-2 application: direct administration of BMP-2 protein and indirect administration by introduction of *BMP-2* gene into target cells. Due to the short half-life of BMP-2 protein, its metabolic clearance *in vivo*, and the need of continuous osteogenic stimulation for efficacious bone regeneration, the former method requires repeat administration or a large initial dose of BMP-2^7^. For example, without a second intervention, up to 12 mg of BMP-2 is needed to heal tibial fractures^6^, which has resulted in unpredictable, severe side effects in some cases, including high rates of nerve root irritation and ectopic bone formation^8^. Such potential risks necessitate the controlled release of BMP-2 or other delivery strategy, such as direct gene transfer of *BMP-2* gene. Gene delivery can be accomplished virally through adeno-associated virus (AAV) and lentivirus, or non-virally in the form of plasmid DNA with different transfection methods^9^. In comparison to direct administration of proteins, *BMP-2* gene transduction into cells presents obvious advantages. For example, BMP-2 protein is produced endogenously by the resident transduced cells; thus, repeat injection or large dose usage of expensive BMP-2 protein is not needed. In addition, the time duration of BMP-2 presence may be controlled by adjusting the expression level of *BMP-2* gene and the type of delivery vectors.

Currently, *ex vivo* gene transfer to produce genetically modified stem cells requires the additional process of *in vitro* culture to achieve gene expression in cells, which is costly and requires cell manipulation. In particular, the additional cell culture step is incompatible with point-of-care treatment. Therefore, scaffold-mediated gene delivery and localized transduction at fracture sites has been proposed as an alternate approach that does not involve *in vitro* cell culture prior to any surgical implantation. Since void- or gap-filling biomaterial scaffolds are normally required for the treatment of large bone defects or nonunion, such scaffolds may be easily adopted as a carrier system for gene vectors. In 1996, Fang *et al*.^10^ reported the use of collagen sponge impregnated with plasmid DNA carrying *BMP-4* gene for the repair of rat bone nonunion, and showed that some of repair cells were transduced by *BMP-4* gene, inducing enhanced bone growth. This landmark work, as well as subsequent work from other groups, have prompted a new “gene-activated matrix/scaffolds” strategy for augmented bone repair^11–15^. However, most of the reported studies have used naked plasmid cDNA with various carriers, which present some advantages such as low immunogenicity and cost-effectiveness, but cellular uptake of these naked DNA is generally of poor efficiency^16^. In particular, plasmid DNAs have low capacity in transducing primary cells such as human mesenchymal stem cells (hMSCs), the most promising cell type for bone regeneration. Highest plasmid DNA transfection efficiency into human bone marrow MSCs (hBMSCs) was reported to be ≤45% by using poly-ethyleneimine as the gene delivery vehicle; however, the process also resulted in more than 20% cell death after 72 hours^17^.

Recently, viral vectors have been used as gene carrier to enhance bone regeneration. For example, self-complementary adeno-associated viral (AAV) constructs encoding BMP-2 were coated on a porous polymer Poly(ε-caprolactone) scaffold and implanted into critically sized immunocompromised rat femoral defects, which were able to transduce hMSCs, leading to significant increases in defect mineral formation as well as mechanical properties^18^. However, the transduction efficiency is relatively low (<10%). In addition, the AAV viral genome was diluted during cell dividing because recombinant AAV genomes were present as extrachromosomal forms^19^. In comparison, lentiviral vector is able to efficiently infect both dividing and non-dividing cells, and has been widely used to achieve stable gene transduction^20^. Although lentiviruses have a risk to integrate into the host cell genome, integration sites may be more restricted in comparison with other viral vectors, thus reducing the risk of insertional mutagenesis^21^. A recent study reported that lentivirus is able to produce up to 90% transduction efficiency in hBMSCs, with a long-term gene expression^22^. Lenti-vector-based genetic engineering of stem cell has been used in clinical trials without evidence of vector-induced genotoxicity or other adverse effects^23, 24^. Therefore, we proposed to develop a single-step procedure to co-introduce lentiviral vector containing *BMP-2* gene, together with hBMSCs, into scaffolds without an *in vitro* pre-transduction process. We hypothesized that the simultaneous packaging of regenerative *BMP-2* gene and hBMSCs would result in localized gene transduction *in situ*, BMP-2 production, robust osteogenesis, and thus, efficient bone regeneration.

**Figure 1:**
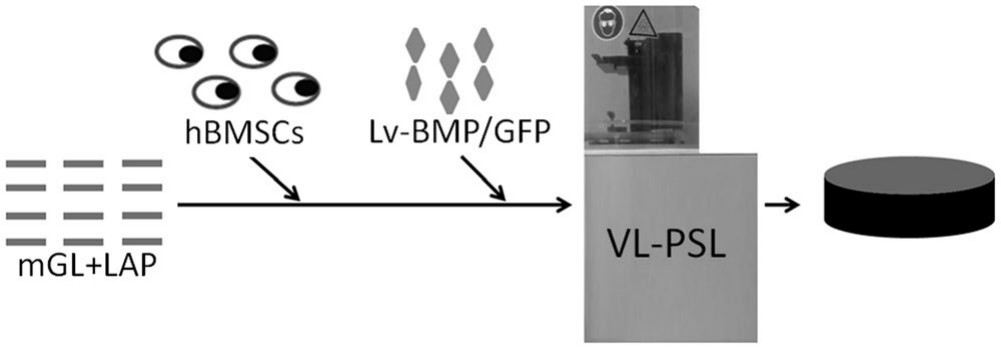
One-step VL-PSL fabrication of engineered bone consisting of photocrosslinked methacrylated gelatin (mGL) encapsulating hBMSCs and lentiviral *BMP-2/GFP* vector constructs (Lv-BMP/GFP).

We have recently developed a novel, visible light-based projection stereolithographic technology (VL-PSL)^25^, for the fabrication of customized scaffolds using natural or synthesized biopolymers with precisely controlled architecture, thus overcoming the challenges of using more conventional methods. In addition, this new VL-PSL procedure allows simultaneous live cell encapsulation, resulting in uniform cellularization throughout the structure of the scaffolds. Therefore, in this study, we applied VL-PSL to produce gene-activated scaffolds by incorporating a lentiviral construct that encodes the human *BMP-2* gene and *green fluorescent protein (GFP) reporter* gene (Lv-BMP/GFP) and osteoprogenitor cells into a photocrosslinkable gelatin (mGL) solution, which then underwent VL-PSL to form a three-dimensional (3D) hydrogel construct (Fig. 1). hBMSCs were used as the osteoprogenitor cells owing to their relatively easy accessibility and high osteogenic capacity upon stimulation by osteoinductive factors such as BMP-2^26^. To our knowledge, this is the first report of the application of 3D printing technology in fabricating gene- and cell-activated bone scaffolds. We first assessed whether VL-PSL is compatible with *in situ* gene delivery in terms of the viability of viral vectors and target cells. We then tested our hypothesis by examining *in situ* transduction efficiency within construct, endogenous BMP-2 production, and bone formation *in vitro* and *in vivo*. Our overall goal was to develop a gene- and cell-activated biomaterial scaffold with precisely controlled architecture and the capability to enhance bone formation for the treatment of bone defects, including nonunion.

## Results

### Efficiency of ex vivo transduction of Lenti-GFP and Lenti-BMP-2 into hBMSCs

In this study, two lentiviral gene constructs were used. The lenti-CMV-BMP-2/GFP (Lv-BMP/GFP) construct contained human cytomegalovirus (CMV) immediate early promoter driven a full length of human *BMP-2* gene (1.2 kB) followed by an internal ribosome entry site (IRES) and a *GFP reporter* gene to allow monitoring of gene transfer efficiency. A lenti-CMV-GFP (Lv-GFP) construct carrying only the *GFP reporter* gene served as the control (see Supplementary Fig. S1A). Using a conventional *ex vivo* transduction procedure on 2D cultures of hBMSCs, green fluorescence was observed as early as 48 hours in either Lv-GFP or Lv-BMP/GFP transduced cells (see Supplementary Fig. S1B). Equal efficiency (~85%) was observed in the two groups. To analyze the osteoinductive effect of the expression of *BMP-2* gene, the activity of alkaline phosphatase (ALP) in hBMSCs, an early marker of osteogenesis, was assessed 7 days post-transduction. High enzymatic activity of ALP, as seen by strong histochemical staining (purple), was observed only in the Lv-BMP/GFP group (see Supplementary Fig. S1C), indicating the functional bioactivity of the BMP-2 produced after gene transduction.

### *In vitro* characterization of constructs produced by single-step fabrication

**Figure 2:**
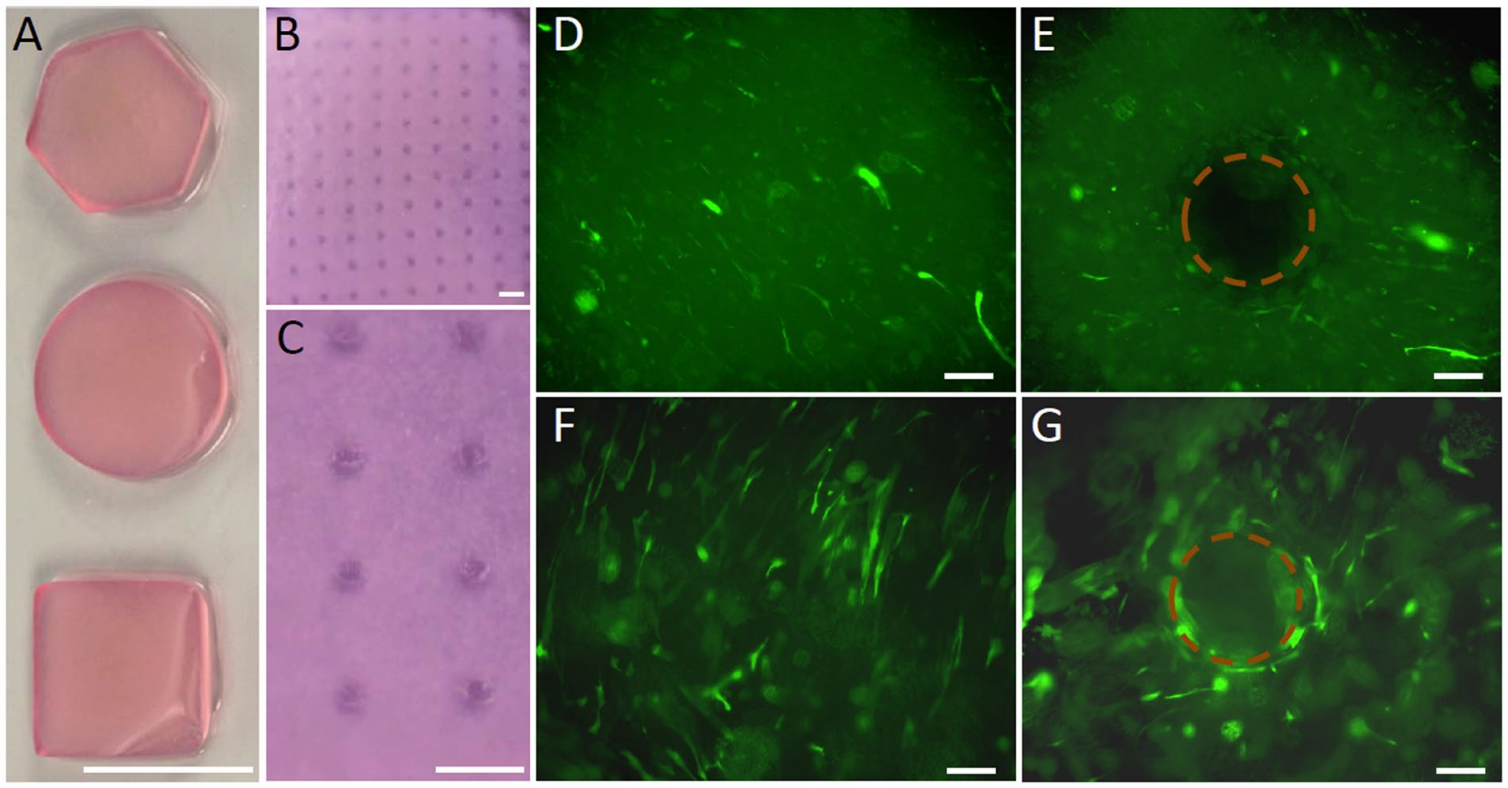
VL-PSL based fabrication of hydrogel scaffolds suitable for cell encapsulation. (**A**) Solid hexagonal, cylindrical and cubic mGL scaffolds or (**B** and **C**) porous scaffolds made with VL-PSL. (**D**–**G**) Lentiviral gene transduced hBMSCs encapsulated within solid (**D**, **F**) or porous (**E**, **G**) hydrogel scaffold. On culture day 2 post fabrication, GFP expression was detected based on fluorescence imaging in constructs from the GFP (**D** and **E**) or BMP (**F** and **G**) groups. Circles in E & G denote the absence of cells in the pores within the scaffold. Bar = 10 mm in A, 2 mm in **B** and **C**, and 200 μm in (**D**–**G**).

hBMSCs were introduced with either Lv-BMP/GFP (BMP group) or Lv-GFP (GFP group) to the mGL solution, and were encapsulated into the hydrogel scaffolds in a single step, using a VL-PSL 3D printing technology previously developed (Fig. 1)^23^. As shown in Fig. 2A,B and C, VL-PSL was able to produce hydrogel constructs with different architectures and porous infrastructures. More importantly, GFP expression was observed in both groups and the transduction efficiency in both groups was similar at ~65–80 percent (Figs 2D,F, S4). We also observed that cell distribution was uniform within the construct and conformed to the scaffold geometry as well. For example, no cells were seen in the pores, (Fig. 2E,G), as designed.

### BMP-2 secretion from constructs in BMP group

**Figure 3:**
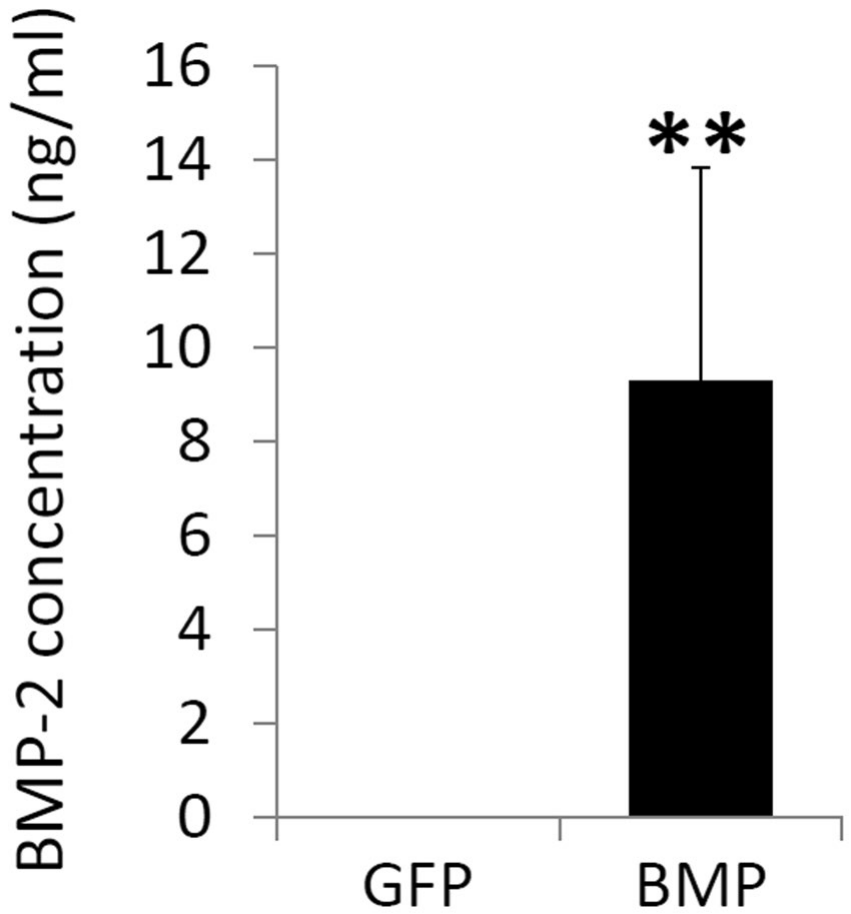
Production of BMP-2 by hBMSCs encapsulated in gene-activated hydrogel scaffolds. Conditioned medium was collected from culture day 4–8 and assayed for BMP-2 by ELISA. The results showed BMP-2 production only in cultures of the BMP group. **p < 0.01.

The expression of the transduced BMP-2 in the 3D constructs was analyzed next. Cell laden scaffolds were cultured in basal growth medium for 4 days, and then the BMP-2 concentration in the medium was measured by enzyme-linked immunosorbent assay (ELISA). In the BMP group, BMP-2 concentrations were at 5–15 ng/ml (Fig. 3), which was within the range of BMP-2 concentrations (~10 ng/ml) often used to induce BMSCs osteogenesis *in vitro* ^27, 28^. In contrast, in the GFP control group, the level of BMP-2 protein was at the undetectable range, which was expected since BMP-2 was not supplemented into the culture medium.

### Robust osteogenesis in BMP group constructs

**Figure 4:**
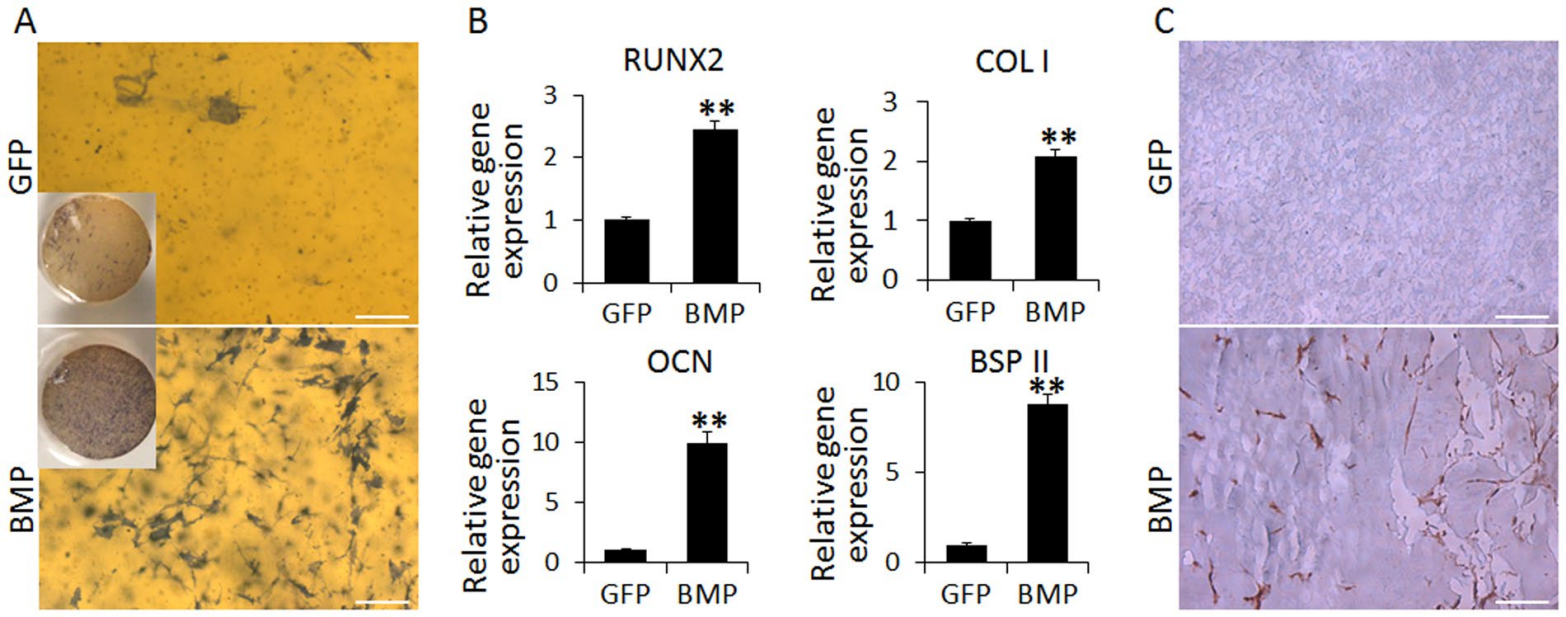
Assessment of osteogenic differentiation of hBMSCs encapsulated in gene-activated hydrogel. (**A**) ALP staining at day 14 post fabrication. Purple staining of ALP activity was only seen in the BMP group. Bar = 100 μm. (**B**) Real-time RT-PCR analysis of expression of osteogenic genes at day 28. All results were expressed relative to the level at day 0. RUNX2, Runt-related transcription factor 2; COL1, collagen type I; OCN, osteocalcin; and BSP2, bone sialoprotein II. Expression of all osteogenesis-associated genes was significantly higher in the BMP group compared to GFP group (**p < 0.01) (**C**) Osteocalcin immunohistochemistry. Brown staining of osteocalcin was detected only in the BMP group. Bar = 100 μm.

After 14 days in culture, the constructs were histochemically stained *in toto* for ALP activity, detectable as purple staining (Fig. 4A). In the BMP group, most cells appeared densely stained throughout the scaffolds, while cells in the GFP control group showed poor staining, clearly indicating the substantially higher activity of ALP in cells of the BMP constructs. After 4 weeks in culture, real time RT-PCR was employed to analyze the expression of osteogenic marker genes in both groups. The expression of *Runt-related transcription factor 2 (RUNX2)*, *Collagen type I (COL 1)*, *Osteocalcin (OCN)* and *Bone sialoprotein (BSPII)* genes were significantly higher in the BMP group, compared to those in GFP control group (Fig. 4B). Osteocalcin immunohistochemistry further confirmed its deposition within the BMP constructs (Fig. 4C). We also examined whether the higher level of extracellular matrix production in the BMP group constructs would contribute to higher mechanical properties. We measured the compressive moduli of scaffolds in both groups at culture day 35, and found that the BMP group constructs became stiffer with time with ~90% higher compressive modulus than those in the GFP control group (see Supplementary Fig. S2). Taken together, these results indicate that hBMSCs encapsulated within the BMP group constructs underwent robust osteogenesis, which was not seen in the GFP group.

### Assessment of *in vivo* ectopic bone formation capacity of VL-PSL fabricated, hBMSC-seeded constructs

**Figure 5:**
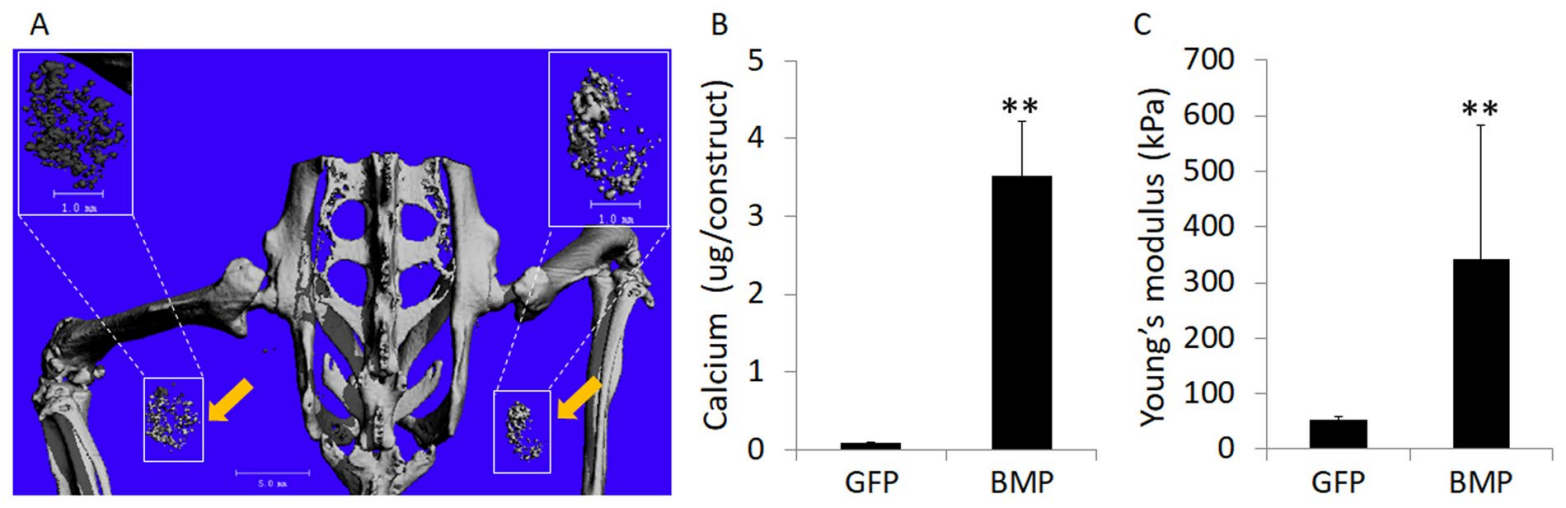
Assessment of ectopic bone formation upon intramuscular implantation of hBMSC-seeded, gene-activated constructs at 3.5 months after implantation. (**A**) µCT imaging of BMP group, showing mineralization sites consistent with ectopic bone formation (indicated with yellow arrows and white rectangular frames). (**B**) Quantitation of calcium deposition in constructs showed significant mineralization only in the BMP group. (**C**) Mechanical testing indicated significantly elevated compressive Young’s modulus in the implanted constructs of the BMP group, suggesting robust bone matrix deposition. **p < 0.01.

After fabrication, the constructs from the BMP and GFP groups were immediately and individually implanted into the hindlimb musculature of SCID mice. At 3.5 months post-implantation, micro-computed tomography (µ-CT) imaging showed detectable mineralized areas in the BMP group, suggesting ectopic bone formation in muscle (Fig. 5A), which were absent in the GFP control implants. The implants were harvested and then processed for biochemical, mechanical, and histological analyses. For histology, the samples were processed without de-calcification. In agreement with the μ-CT imaging results, the total calcium content of the BMP group was 3.6 μg/construct, substantially higher than that of the GFP group (0.08 μg/construct; Fig. 5B, p < 0.01). Constructs in the BMP group also exhibited stronger mechanical property with a compressive modulus of 350 kPa (Fig. 5C, p < 0.01), which is significantly higher than the constructs right before implantation (35kPa). Such enhancement could have resulted from the higher content of calcium and extracellular matrix. In comparison, the stiffness of the GFP group implants was low and unchanged with time.

**Figure 6:**
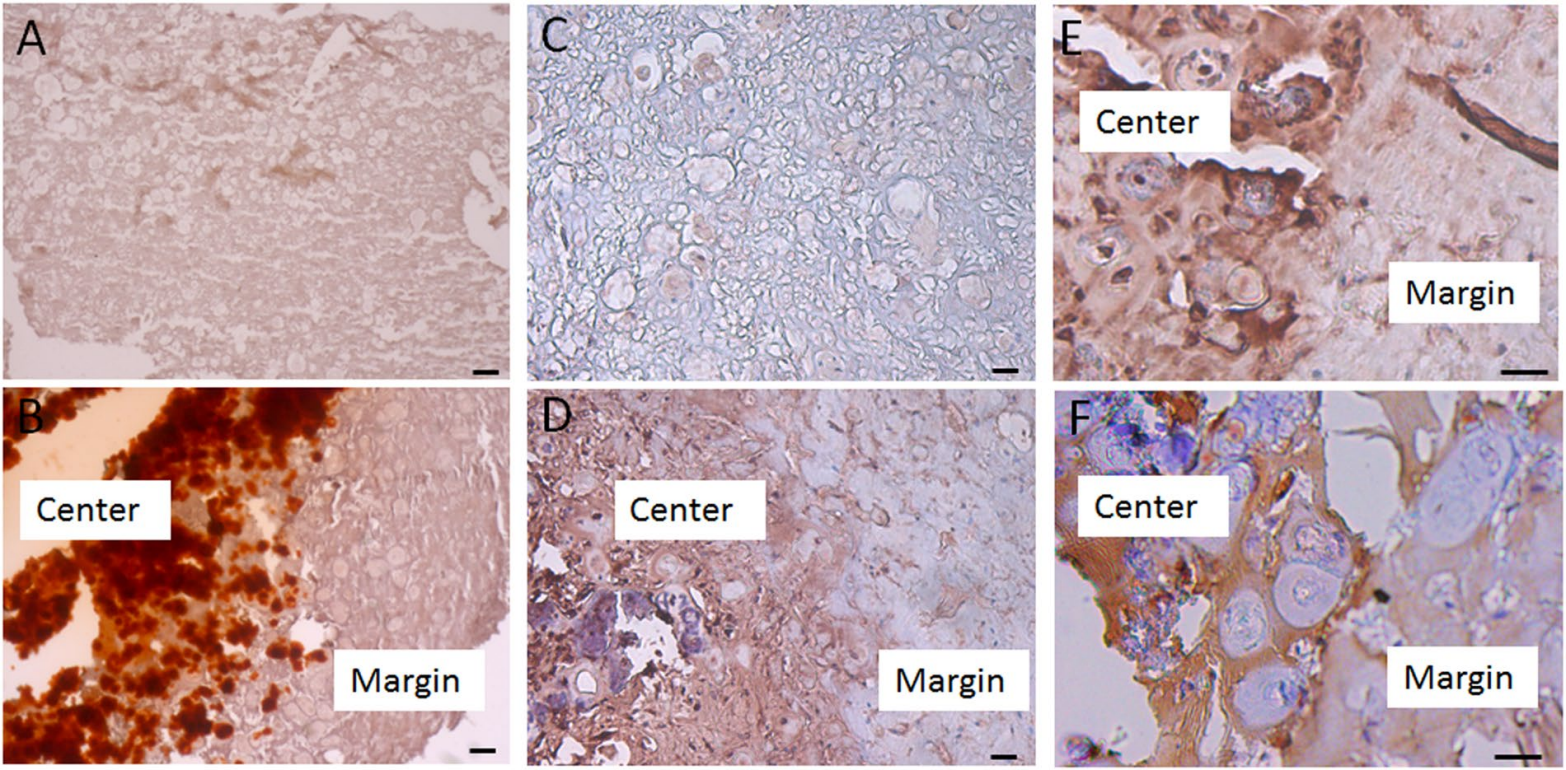
Histological and immunohistochemical examination of ectopic bone formation in intramuscular implants of hBMSC-seeded, gene-activated constructs at 3.5 months post-implantation. (**A**,**B**) Alizarin red staining revealed abundant mineralization in (**B**) BMP group, but absent in (**A**) GFP group. (**C**, **D**,**E**) Osteocalcin immunohistochemistry detected absence of staining in (**C**) GFP group, but positive staining in (**D**,**E**) BMP group, specifically in the center of the implant, compared to the margin. (**F**) GFP immunohistochemistry showing localization of gene-transduced hBMSCs primarily at the center of the implant, ﻿coinciding with the expression of osteocalcin. Bar = 100 μm.

As shown in Fig. 6, there was no apparent sign of host tissue reaction, indicated by minimal ingrowth of host tissue and blood vessels, which was further confirmed by H&E staining (see Supplementary Fig. S3). Alizarin red staining was employed to assess the spatial distribution of tissue mineralization, and the results showed that robust positive staining (red) was only seen in the BMP group (Fig. 6A,B), in agreement with the results from μ-CT imaging. It is noteworthy that calcium deposition was localized only to the center of the construct rather than its margin, suggesting localized bone formation within the implant without outgrowth or contribution from host cells.

To assess whether the mineralization observed was a result of genuine osteogenic differentiation and ossification, immunohistochemistry was performed to detect osteocalcin, a bone cell specific marker. Positive osteocalcin staining was clearly seen only in the BMP group (Fig. 6D,E), but not in the GFP group (Fig. 6C), indicating osteogenesis and the presence of osteoblasts within the *BMP-2* gene-loaded constructs.

The fact that osteogenesis was only observed within the center area of the implanted constructs (Fig. 6B,D,E) suggested that the transplanted hBMSCs were likely to be the key contributing cell population in this process. This was examined by exploiting the presence of the GFP reporter gene in the lentiviral *BMP-2* gene construct (see Supplementary Fig. S1), resulting in expression of both GFP and BMP-2, and allowing the tracing of the transduced cells using GFP as a marker. We then performed GFP immunohistochemistry to trace the distribution of the transduced hBMSCs. As shown in Fig. 6F, the peripheral area of the constructs showed very weak GFP staining, while the most GFP-positive cells were observed in the center of the constructs, indicating either a higher level of gene transduction or abundance of transduced cells, or both, in the center. This distribution of GFP staining roughly coincided with that of osteocalcin immunostaining and alizarin red positive areas, strongly suggesting that the implanted hBMSCs, not host cells, were the major contributing cell population to osteogenesis and bone formation in the implanted constructs *in vivo*.

## Discussion

Our study reported here has critically extended the potential of gene-activated matrix as a bone repair methodology by developing a gene- and cell-activated customized scaffold, produced by combining a rapid 3D printing technology with viral vector-based gene delivery and stem cell based tissue engineering through a single-step procedure.

Gene-activated matrices have been studied extensively since the first report about two decades ago^10^. In most subsequent studies, the purpose of gene delivery was to release gene harboring vectors from the matrix to transduce host cells. The advantages and disadvantages of this strategy, for example, for bone formation, are obvious. The method is simple and does not require cell transplantation, and *in vitro* cell harvest and additional culture process are not necessary. However, since host cells need to migrate into the scaffold from the surrounding tissues to allow gene transduction, the introduced gene constructs are likely to diffuse out of the scaffold during this time, resulting in out-of-scaffold gene delivery and transgene (e.g., *BMP-2*) expression, and finally, the unregulated bone formation^29^. In addition, the gene transduction and osteogenesis that starts from the edge or margin of the implants may result in reduced or delayed bone formation in the center area, a complication often observed in bone nonunion. Therefore, for effective application of gene-activated matrices for bone formation, it is important to control the amount and location of the expressed BMP-2 and the newly formed bone. Controlled release of gene constructs has been applied, but did not solve the problem completely^30^. To address these requirements, we have designed a method for *in situ* scaffold-mediated stem cell engineering by transplanting progenitor cells simultaneously with desired viral gene vectors, thus allowing *in situ* uptake of virus and gene transfer to control transduction within the scaffold structure, limiting vector release and undesirable bone formation outside of the implanted graft.

The engineered biomaterial scaffold architecture is known to play an important role in modulating bone regeneration and determining the final anatomical aspects of the repaired bone^31^. For example, a 0°/90° alignment of the scaffold fibers demonstrated a significantly improved bone regeneration^32, 33^. Thus, a scaffold fabrication method with customizable capacity is desirable. Currently, there are several different solid freeform fabrication technologies (3D printing) that allow for precise scaffold production based on computer-aided design (CAD) models or clinical imaging. However, not all of them support live cell incorporation. In addition, due to the known sensitivity of DNA to ultraviolet (UV) illumination, i.e., DNA damage resulting from UV-initiated pyrimidine dimerization, fabrication methods using UV as the polymerization trigger are, in principle, incompatible with DNA-based matrix activation. The VL-PSL method employed here utilizes visible light^23^, thus removing the risk of compromising cell viability^25, 34, 35^, or damaging the activity of the viral vectors; this is confirmed by our results showing the viability and infectivity of viral vectors after the VL-PSL process.

To further control a burst release of viral vectors from the biomaterial, a hydrogel scaffold with nano-pores is desired to reduce free diffusion, compared to other scaffolds with larger pores^30, 36^. For this purpose, we have chosen gelatin hydrogel, fabricated by VL-PSL, as the biomaterial because of its biocompatibility and osteoconductive capacity^37–39^. It is also biodegradable, therefore allowing tissue remodeling for new bone formation. A limitation of gelatin is its low mechanical strength (~30 kPa compressive modulus), although robust osteogenesis may compensate in part for this insufficiency, since enhancement of the mechanical strength of the gelatin constructs was observed in the BMP group in our *in vivo* study (Fig. 5D). Recently, we have incorporated poly-D,L-lactic acid/polyethylene glycol/poly-D,L-lactic acid (PDLLA-PEG) in VL-PSL for live cell fabrication, and produced constructs exhibiting much stronger mechanical property (>800 kPa compressive modulus)^35^. However, whether such gel scaffolds support osteogenesis is unknown and will be tested in our future work.

Although lentiviral vectors are not completely devoid of the risk of oncogenicity, no apparent adverse effects with the use of lentiviral vectors have been reported in clinical trials^23, 24, 40, 41^. In addition, lentiviral vectors are highly efficient in transducing primary hBMSCs in comparison to AAV^18, 42^. In our study, the average level of BMP-2 production was 10 ng/ml from 1 × 10^6^ cells in 5 days, which is sufficient to induce hBMSC osteogenesis. In comparison, a study using AAV as the carrier reported a highest BMP-2 concentration at 2 ng/ml^18^. We are unable to identify directly comparable studies, but one group using goat BMSCs and non-viral BMP-2 vectors reported 8 ng/ml BMP-2 production from 1 × 10^6^ cells in 14 days^43^, illustrating the high transfection efficiency of our method. Using our method, after 3 months of culture, BMP-2 production by hBMSCs is still detectable, although the level has decreased to 1.3 ng/ml/1 × 10^6^ cells.

In previous reports using scaffolds loaded with BMP-2 protein or BMP-2 expressing cells, the majority of bone formation is often occurs at the margin areas; in these instances, the location of the excessive bone formation may function as a physical barrier to delay or impair the ossification process in the interior regions of the grafts^44, 45^. In the current *in vitro* study, uniform bone formation (indicated by ALP staining) throughout the construct was observed, representing a more desirable process of bone formation. However, it is interesting to note that our *in vivo* results show higher level of osteogenesis and GFP immunostaining localized at the center instead of at the margin of the implanted construct, which could have resulted from the difference between *in vitro* and *in vivo* culture conditions. In the *in vitro* study, the released virus stayed in the medium, resulting in saturated viral concentration in the margin, sufficient to infect the remaining cells. On the other hand, under *in vivo* conditions, the released virus may be quickly cleared by the body, leading to continuous release of AAV from the implant and less bone formation at the margin. Also, since no observable bone formation is seen outside of the scaffold implants, it is reasonable to assume that the amount of released lentiviral particles is insufficient to transduce the host cells surrounding the implant. Our recent *in vitro* results obtained a Transwell co-culture set-up show only limited gene transduction of cells after being exposed to the conditioned medium of GFP or BMP-loaded scaffolds (*data not shown*); effective gene transduction of hBMSCs only takes place in-scaffold, likely because of the dependence on a sufficient level viral gene construct. In addition, we observe similar spatial distribution of Alizarin red and GFP positive area, indicating that the formation of new bone involves primarily transplanted cells. Taken together, we conclude that the bulk of the viral load is locally transduced into the seeded hBMSCs within the scaffold, without burst release from the scaffolds, which reduces the risk of uncontrolled bone formation. Therefore, the gene and cell-activated construct developed here is a promising and likely safe method to achieve localized and homogeneous bone formation.

It should be pointed out that the *in vivo* bone formation observed here is relatively slow with little vascularization (Figs 6 and S3), which could impair long-term integration between the neo-bone graft and host tissue. In our previous study, we have shown that angiogenesis resulting from viral-vector-based vascular endothelial growth factor (VEGF) transduction is important for enhancement of the regenerative potential of stem cells^46^. A possible reason is that the gene-activated scaffold is applied without the continuous presence of agents, such as Polybrene^47^, that are commonly used to enhance virus absorption and gene transfer efficiency, which may present potential safety risks for clinical application^48, 49^. As a result, we did not achieve a level of BMP-2 production similar to that *in vitro*; future optimization of viral load and judicious formulation of virus adsorption enhancing cocktails may accelerate this process. The paucity of blood vessels in the constructs, which would limit host cell invasion, may be addressed in the future by co-introducing viral gene constructs of other osteoinductive factors, such as VEGF, together with BMP-2^50^.

## Conclusions

We have developed a single-step, *BMP-2* gene-activated, live cell encapsulated scaffold fabrication procedure for bone formation. Efficient transgene expression (*GFP* and *BMP-2*) is seen in hBMSCs encapsulated in the engineered scaffolds. Cell distribution is uniform within the construct and conforms to scaffold geometry. Results from ELISA, real time RT-PCR, immunohistochemistry, and mechanical testing all indicate robust osteogenesis within constructs activated with lentiviral BMP-2 vector, without the supplementation of exogenous BMP-2 protein. After intramuscular implantation in SCID mice, μ-CT imaging showed detectable mineralized areas in the BMP group, suggesting effective ectopic bone formation *in vivo*, which is absent in the GFP control group. In addition, formation of the new bone is localized and restricted within the implanted scaffolds and is primarily associated with the transplanted GFP-labeled hBMSCs, based on coincident staining of osteocalcin and GFP.

## Materials and Methods

### Materials

All reagents used in this study were purchased from Sigma-Aldrich (San Luis, MO), unless otherwise stated.

### Isolation and expansion of hBMSCs

hBMSCs were isolated from bone marrow aspirates obtained during total joint arthroplasty using a previous protocol established in our laboratory^51^ with IRB approval and informed patient consent (University of Washington and University of Pittsburgh). The stemness of hBMSCs was tested by colony forming efficiency and mesoderm differentiation capability (osteogenesis, chondrogenesis, and adipogenesis, *data not shown*). Passage 3 (P3) hBMSCs were used in this study by pooling cells from 3 patients (55-year-old male, 67-year-old female, and 81-year-old male).

### Lentiviral vector preparation

The lenti-CMV-BMP-2/GFP (Lv-BMP/GFP) construct contains human cytomegalovirus (CMV) immediate early promoter driven full length human *BMP-2* gene (1.2 kB) followed by an internal ribosome entry site (IRES) and a GFP reporter gene to allow monitoring of gene transfer efficiency by green fluorescence. A lenti-CMV-GFP (Lv-GFP) construct carrying only the GFP reporter gene served as control (see Supplementary Fig. S1A). Lenti-viral vectors were produced according to the standard protocol^52^.

### Functional testing of lentiviral BMP-2 construct by ex vivo transduction into hBMSCs

P3 hBMSCs were expanded to 80% confluency and the medium was switched to serum free DMEM medium containing lentiviral vectors (total 2 × 10^6^). Sixteen hours after infection, the culture medium was replaced with fresh growth medium (GM, DMEM supplemented with 10% fetal bovine serum, FBS). GFP expression was observed and imaged using an epifluorescence microscope (CKX41, Olympus, Japan) equipped with a color camera (DFC321, Leica, Germany) at 48 hours post-infection. Expression of alkaline phosphatase (ALP) in hBMSCs was qualitatively analyzed at 14 days after infection by ALP histochemical staining using a Leukocyte Alkaline Phosphatase Kit (86 R, Sigma) following the manufacturer’s protocol. Briefly, the culture medium was removed and staining solution was added and incubated with cells at 37 °C for 1 hour. Stained cultures were observed and imaged using a DSL camera (T3i, Canon, Japan).

### Preparation of materials for visible light-based stereolithography (VL-PSL)

Methacrylated gelatin (mGL), serving as the monomer for VL-PSL, was prepared as described previously^39^. Briefly, 3% aqueous gelatin solution was reacted with methacrylic anhydride for 16 hours, dialyzed extensively against H_2_O to remove unreacted reagents, lyophilized, and kept in dark until use. The visible light activated photoinitiator, lithium phenyl-2,4,6-trimethylbenzoylphosphinate (LAP), was synthesized by reacting dimethyl phenylphosphonite (Acros Organics, New Jersey) with 2,4,6-trimethylbenzoyl chloride via a Michaelis-Arbuzov reaction^53^.

### Single step VL-PSL

A customized stereolithographic apparatus (EnvisionTec, Germany) was used for VL-PSL using parameters optimized in our previous study^25^. 10% mGL (w/v) and 0.6% LAP (w/v) were dissolved in 30 ml HBSS (Ca & Mg+) completely and adjusted to pH 7.4 with sodium hydroxide. Insulin-Transferrin-Selenium (ITS - 10 mg/L insulin, 5.5 mg/L transferrin, and 6.7 μg/ml sodium selenite; Invitrogen) and Polybrene (8 µg/mL) were added to maintain cell viability and virus infection ability, respectively, under serum free condition. hBMSCs (5 × 10^6^ cells/ml final cell density) were then mixed into the gelatin solution together with lenti-GFP or lenti-BMP-2/GFP viral constructs (1 × 10^8^ for each).

The cell suspension was poured onto the plate and VL-PSL started using a procedure that we have previously established^25^. Solid hexagonal, cylindrical, and cubic models or a porous cylindrical model (with 500 μm diameter tubes passing top-to-bottom through the structure and 2 mm distance between the closest edges of any two tubes) were used as the templates for fabrication. To maintain consistency in quantitative assays, a solid cylindrical 3D model (5 mm diameter and 2 mm height) was used to make constructs for all subsequent experiments. After VL-PSL, the solidified scaffolds were taken off the platform and transferred to growth medium (10% FBS), supplemented with Polybrene, but without antibiotics for 4 hours. Afterwards, the medium was removed and replaced with fresh, full growth medium. The medium was changed every 3 days, up to 35 days. The constructs containing lenti-GFP or lenti-BMP-2/ GFP vectors were named as the GFP group or BMP group, respectively.

Gene transfer efficiency within the 3D construct was assessed after 2 days on the basis of GFP fluorescence. Microscopic fields obtained by fluorescence (GFP) and phase contrast imaging were merged to determine infection efficiency (see Supplementary Fig. S4). Specifically, the number of fluorescent, GFP-positive cells detected by fluorescence microscopy was expressed as a percentage of the total number of cells detected by phase contrast microscopy.

### BMP-2 quantitation

Cylindrical constructs (5 mm diameter and 2 mm height) were individually cultured in 200 µl GM. On day 4, the medium was replaced with fresh GM and constructs were cultured for 4 additional days. BMP-2 accumulation in the culture medium during this period, produced by the transduced hBMSCs, was quantified using a BMP-2 Quantikine ELISA Kit (R&D, Minneapolis, MN).

### Alkaline phosphatase (ALP) staining

At day 14 post-fabrication, ALP expression in hBMSCs encapsulated within the gelatin constructs were histochemically assessed as described above. Briefly, the cultured constructs were removed from culture medium and immersed into the staining solution and incubated at 37 °C for 1 hour. The macroscopic and microscopic appearance of stained constructs was imaged using a DSL camera (T3i, Canon, Japan) or a microscope (CKX41, Olympus, Japan) equipped with a color camera (DFC321, Leica, Germany).

### Real-time quantitative reverse transcription polymerase chain reaction (real time RT-PCR)

Expression of osteogenic genes in hBMSCs was analyzed with real time RT-PCR after 28 days *in vitro* culture. Constructs from GFP or BMP groups were washed 3 times with HBSS, placed into Trizol (Invitrogen), and ground with a plastic pestle. Total RNA was extracted into the aqueous layer after adding chloroform and purified using an RNeasy Plus Mini Kit (Qiagen, Maryland). Real time RT-PCR was performed using the SYBR Green-based method and a real-time PCR system (StepOne Plus, Applied Biosystems). Expression levels of osteogenesis-related genes such as Runt-related transcription factor 2 (RUNX2), collagen type I (COL1), osteocalcin (OCN), and bone sialoprotein II (BSP2) were analyzed by the 2^−ΔΔct^ method with 18 S rRNA as the control housekeeping gene.

### Histological analysis

Samples were fixed in 10% buffered formaldehyde (Fisher Chemicals, Fari Lawn, NJ), dehydrated in a series of ethanol baths (70, 95 and 100%) and finally in xylene, and embedded in paraffin. Tissue blocks were sectioned at 6 μm thickness using a standard microtome (Leica Biosystems, France), which were then subjected to standard H&E staining and imaged with a CKX41 microscope (Olympus, Japan) equipped with a Leica DFC 3200 camera.

### Osteocalcin immunohistochemistry

The extent of osteogenesis was further assessed based on immunohistochemical staining of osteocalcin. Briefly, histological sections were subjected to antigen retrieval by treatment with chondroitinase ABC (100 mU/ml) and hyaluronidase (250 U/ml), peroxidase blocking in 3% H_2_O_2_, and further blocked in 1% horse serum. Primary antibody (rabbit anti-osteocalcin; abcam ab93876, 1:200) was applied to the section, followed by overnight incubation at 4 °C. Detection of primary antibody was performed with biotinylated goat anti-rabbit antibodies (Vector Laboratories, Burlingame, CA). Secondary peroxidase-conjugated antibodies were visualized using a Vectorstain Elite ABC and developed with the VIP kit (Vector Laboratories). Sections were counterstained with hematoxylin prior to mounting. Images were captured with a CKX41 microscope (Olympus, Japan) equipped with a Leica DFC 3200 camera.

### Mechanical testing

At culture day 35, the compressive moduli of constructs in the GFP and BMP groups were assessed using a mechanical tester (ElectroForce 3200, Bose). Constructs were placed between two metal plates and compressed to 20% of original thickness (0.4 mm) with the force recorded. Compressive Young’s modulus was determined from the linear area in the stress/strain curve.

### Intramuscular implantation of constructs

Female Severe Combined Immunodeficiency (CB17/Icr-*Prkdcscid*/IcrIcoCrl SCID^®^) mice (8–12 weeks old; Charles River Laboratories; Wilmington, MA) were used according to University of Pittsburgh Institutional Animal Care and Use Committee (IACUC) approved protocols. All experiments were performed in accordance with relevant guidelines and regulations. Prior to surgery, the hind legs of the animals were shaved on both sides and cleansed with alcohol. A skin incision (around 6 mm long) was made to reveal the muscles of the upper long bone. Blunt dissection of the muscles was carefully performed to create pockets for the constructs without making traumatic damage to the muscle tissues. Constructs from the GFP or BMP group (n = 6 for each group) were then inserted into the muscle on both sides of the hind legs. Polybrene was not included for the *in vivo* study due to potential safety issue in the future clinical application^48, 49^. The muscles and skin of the animals were then individually and sequentially sutured.

### Micro–computed tomography (µCT)

At 3.5 months after implantation, the mice were anesthetized and scanned at 14 μm resolution with a commercial μ-CT system (Scanco vivaCT 40, Switzerland). Three-dimensional reconstructions of detectable mineralized tissue in implantation site were created at 29 μm resolution for analytical visualization. A volume of interest was defined for each specimen, and a threshold was chosen to exclude any non-mineralized tissue. The total volume and density of bone was then determined.

### Measurement of total calcium content

The animals were sacrificed at 3.5 months post-implantation and the implants were harvested. After clearing of adventitious muscle tissues, the constructs were soaked in 1 ml 1 N HCl and ground thoroughly. After extraction for 3 days at room temperature, the solution was cleared by centrifugation at 12,000 g for 5 minutes and the calcium concentration was measured using a calcium colorimetric assay kit (BioVision, San Francisco, CA), and total calcium deposition in each construct was calculated.

### Alizarin red staining

Calcium deposition in the implants was further examined histologically by Alizarin Red S staining (Rowley Biochemical, Danvers, MA) and then imaged with a CKX41 microscope (Olympus, Japan) equipped with a Leica DFC 3200 camera.

### GFP Immunohistochemistry

To analyze the distribution of implanted hBMSCs, on the basis of their transduction by lentiviral vectors containing GFP, immunohistochemistry was performed using rabbit antibodies directed against GFP (Abcam, ab6556; 1:500) using the procedure described above for osteocalcin.

### Statistics

All experiments were performed in triplicates. All results are presented as means ± standard deviation and analyzed using paired *t*-test with statistical significance set at p-value < 0.05. In all cases, * was used for p < 0.05, and ** for p < 0.01.

### Data availability

The datasets generated from the current study are available from the corresponding author upon reasonable request.

## Electronic supplementary material

Supplementary Figures
