# Supplementary material for: Projection Stereolithographic Fabrication of BMP-2 Gene-activated Matrix for Bone Tissue Engineering
Source: Sci Rep. 2017 Sep 12;7:11327. doi: 10.1038/s41598-017-11051-0 (PMC5595921; doi:10.1038/s41598-017-11051-0)
Supplement: Supplementary file 1 — Supplementary Figures [file 41598_2017_11051_MOESM1_ESM.pdf]

Projection Stereolithographic Fabrication of BMP-2 Gene-activated Matrix for Bone Tissue Engineering

Hang Lin, Ying Tang, Thomas P. Lozito, Nicholas Oyster, Robert B. Kang,
Madalyn R. Fritch, Bing Wang, and Rocky S. Tuan

Supplementary Figure S1. Design and functional testing of lentiviral constructs used for gene-activation of hydrogel scaffold. (A) Two lentiviral vector constructs were used, Lv-BMP/GFP and Lv/GFP, both containing a GFP reporter gene. Whole BMP-2 gene was used when preparing the construct of Lv-BMP/GFP. (B) hBMSCs showed positive GFP expression at 2 days post-infection with either lenti-BMP-2 or lenti-GFP, observed and imaged with a fluorescence microscope. Bar = 200 μm. (C) ALP histochemistry showed positive staining only in lenti-BMP-2 transduced hBMSCs at day 7 post-infection.

Supplementary Figure S2. Determination of compressive Young's moduli of cultured hBMSC-seeded, gene-activated constructs from GFP or BMP group on culture days 1 and 35. *, $p<0.05$; **, $p<0.01$.

Supplementary Figure S3. Histological examination (H&E staining) of ectopic bone formation in intramuscular implants of hBMSC-seeded, gene-activated constructs at 3.5 months post-implantation. Bar = 100 μm.

Supplementary Figure S4. Gene transfer efficiency within the 3D construct was counted at Day 2 after fabrication. Microscopic fields obtained by (A) fluorescence (GFP) and (B) phase contrast imaging were merged to determine infection efficiency. Gene transfer efficiency is calculated as the number of fluorescent, GFP-positive cells expressed as a percentage of the total number of cells.

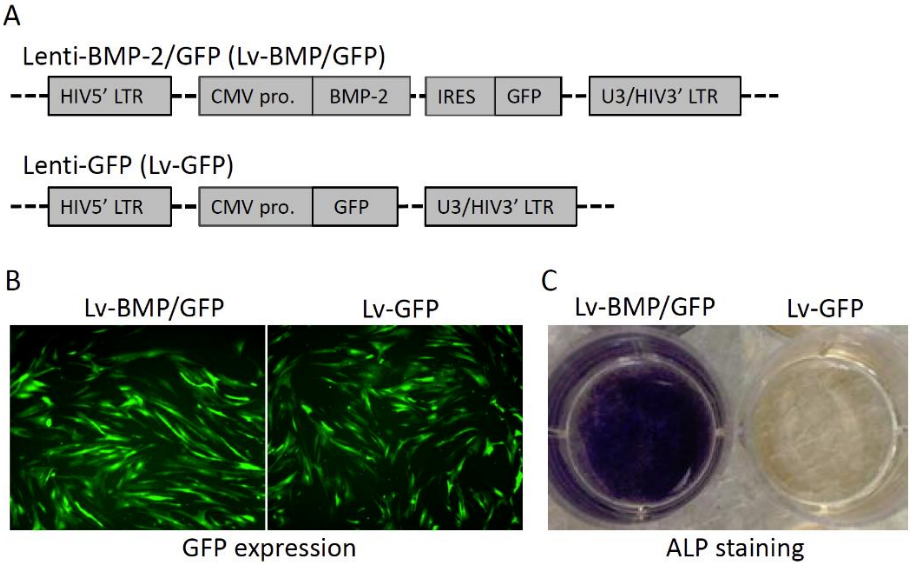

Supplementary Figure S1. Design and functional testing of lentiviral constructs used for gene-activation of hydrogel scaffold. (A) Two lentiviral vector constructs were used, Lv-BMP/GFP and Lv/GFP, both containing a GFP reporter gene. Whole BMP-2 gene was used when preparing the construct of Lv-BMP/GFP. (B) hBMSCs showed positive GFP expression at 2 days post-infection with either lenti-BMP-2 or lenti-GFP, observed and imaged with a fluorescence microscope. Bar = 200 µm. (C) ALP histochemistry showed positive staining only in lenti-BMP-2 transduced hBMSCs at day 7 post-infection.

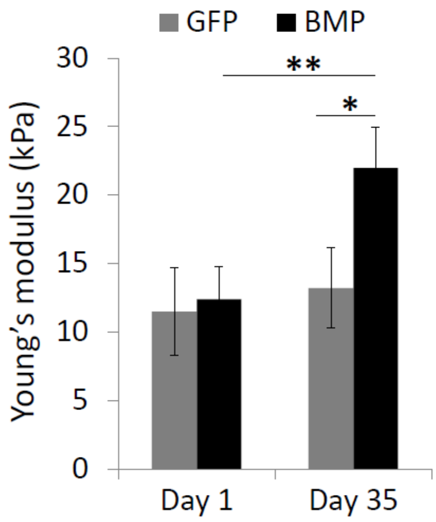

Supplementary Figure S2. Determination of compressive Young's moduli of cultured hBMSC-seeded, gene-activated constructs from GFP or BMP group on culture days 1 and 35. *, $p<0.05$; **, $p<0.01$.

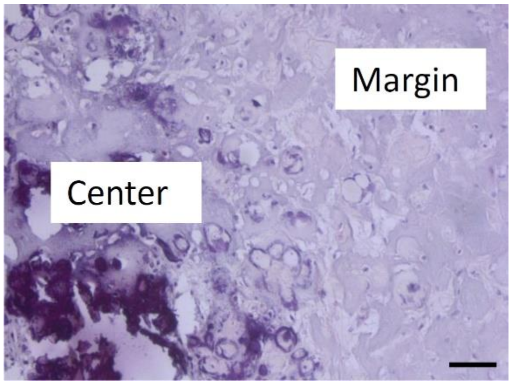

Supplementary Figure S3. Histological examination (H&E staining) of ectopic bone formation in intramuscular implants of hBMSC-seeded, gene-activated constructs at 3.5 months post-implantation. Bar = 100 µm.

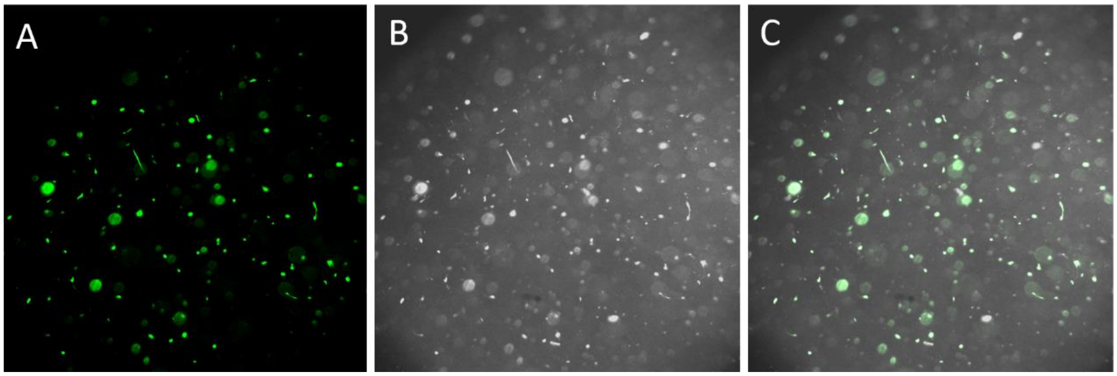

Supplementary Figure S4. Gene transfer efficiency within the 3D construct was counted at Day 2 after fabrication. Microscopic fields obtained by (A) fluorescence (GFP) and (B) phase contrast imaging were merged to determine infection efficiency. Gene transfer efficiency is calculated as the number of fluorescent, GFP-positive cells expressed as a percentage of the total number of cells.
